# Effects of web-based mindfulness training on psychological outcomes, attention, and neuroplasticity

**DOI:** 10.1038/s41598-023-48706-0

**Published:** 2023-12-19

**Authors:** María Guadalupe Mora Álvarez, Britta Karen Hölzel, Benno Bremer, Maximilian Wilhelm, Elena Hell, Ebru Ecem Tavacioglu, Kathrin Koch, Alyssa Torske

**Affiliations:** 1 Department of Diagnostic and Interventional Neuroradiology, School of Medicine, Technical University of Munich, Munich, Germany; 2 TUM-Neuroimaging Center (TUM-NIC), Klinikum rechts der Isar, Technical University of Munich, Munich, Germany; 3 Department of Neurology, Charité – Universitätsmedizin Berlin, corporate member of Freie Universität Berlin and Humboldt-Universität zu Berlin, Berlin, Germany; 4 Center for Psychotherapy Research, Heidelberg University Hospital, Heidelberg, Germany; 5 Department of Psychology, Ludwig Maximilians Universität München, Munich, Germany; 6 Department of Psychology, University of Würzburg, Würzburg, Germany; 7 Graduate School of Systemic Neurosciences, Ludwig Maximilians Universität München, Martinsried, Germany

**Keywords:** Neuroscience, Psychology, Health care, Medical research

## Abstract

Mindfulness meditation training (MMT) reliably reduces stress and anxiety while also improving attention. The primary aim of this study was to investigate the relationship between MMT, stress and anxiety reduction, and its impact upon improvements in attention on the behavioral and neuronal levels. As a second aim, we sought to explore any relationship between MMT, attention, and modified states of mind such as flow. 118 healthy, meditation-naïve, participants were either assigned to a 31-day, web-based, MMT or an active control, health training (HT). Participants underwent functional magnetic resonance imaging while performing the attention network test (ANT) to assess functional and behavioural attentional changes, diffusion tensor imaging (DTI) to assess microstructural neuronal changes and completed relevant questionnaires to explore changes in psychological outcomes. Results confirmed a reduction in perceived stress and anxiety levels in the MMT group and significant improvements in the overall reaction time during the ANT, albeit no specific effects on the attentional components were observed. No statistically significant changes were found in the HT group. Interestingly, a significant group-by-time interaction was seen in flow experience. Functional data exhibited an increased activity in the superior frontal gyrus, posterior cingulate cortex, and right hippocampus during the alerting condition of the ANT after the MMT; decreased stress and trait anxiety were significantly correlated with the activation in the right hippocampus, and increased flow was also significantly correlated with all the aforementioned areas. DTI data showed increased fractional anisotropy values in the right uncinate fasciculus indicating white matter microarchitecture improvement between the right hippocampus and frontal areas of the brain. This study, therefore, demonstrates the effectiveness of web-based MMT on overall well-being and attentional performance, while also providing insight into the relationship between psychological outcomes, attention, and neuroplastic changes.

## Introduction

Mindfulness meditation is defined as present-centered awareness of thoughts, feelings, or sensations acknowledged and accepted free of judgment^1^. In this paper, we used the term mindfulness meditation training (MMT) to refer to any program or training that incorporates mindfulness meditation in their practice. MMT has been demonstrated to notably improve perceived stress^2–5^, anxiety, and consequently mental health^2,6–9^, while also playing a role in improving various domains of cognitive function including attention, working memory, and problem-solving skills^10–13^. On the other hand, prolonged periods of stress and anxiety can elicit a wide range of physical and psychiatric diseases^14^, showing detrimental effects on cognitive performance^15–19^. One of the main cognitive processes affected by chronic stress and anxiety is attention^18,20^. Attention is generally defined as an essential domain of cognitive functioning that allows for the selection of stimuli for further processing^21^. While acute stress, from an evolutionary standpoint, plays a crucial role in drawing attention to threatening stimuli, chronic or disordered stress can have a negative effect on the ability to distribute attentional resources efficiently, thereby implicating cognitive functioning^22^. An additional adaptive response to threats in the environment is anxiety. Like stress, anxiety can become maladaptive in trait anxious individuals causing deficits in cognitive functioning through the similar, inefficient, distribution of attentional resources^18^. While stress and anxiety have been demonstrated to have a negative impact on cognitive, or attentional, functioning as a whole^15–18^, a recent meta-analysis conducted on MMT was able to demonstrate that MMT can, overall, have a positive influence on attentional mechanisms^23^. Given the documented detrimental effects of stress and anxiety on cognitive function and the positive effects of MMT on attention, we investigated whether stress and anxiety reduction following mindfulness training would be related to improvements in attention both on the behavioral and neuronal level.

To examine the effects of MMT on the different components of attention, this work followed Michael Posner’s attention network model which encompasses three attentional mechanisms that rely on specific, separate, networks in the brain: Alerting (i.e., vigilance), orienting (i.e., selection), and executive control (i.e., the process of blocking distractors while performing a task)^24–27^. These attentional mechanisms can each be measured via the attention network test (ANT)^28^. Posner’s ANT was therefore utilized in the present study to investigate: a) the effects of a web-based MMT on the different attentional components and psychological outcomes, b) any link between psychological outcomes and improved attention, and c) brain functional and anatomical changes.

Not only was previous research able to determine MMT’s effects on attentional mechanisms, but it was also demonstrated to have a positive effect on the psychological flow experience^29^. The concept of flow was introduced by the Hungarian psychologist, Mihaly Csikszentmihalyi, where he defined flow as a state of consciousness of optimal concentration and absorption in a given task which leads to a state of satisfaction or optimal experience^30^. Though research on flow is nascent, a recent study showed that an increase in flow experience has a positive impact on the ability to sustain attention^31^. Given the positive effect of MMT on flow and the close relationship between flow and attention, we thought that it would be interesting to investigate the association between MMT, flow-experience, and attention, in addition to how the reduction of stress and anxiety could lead to improvements in attention. To elucidate these associations, we used magnetic resonance (MR) neuroimaging techniques, in addition to standardized questionnaires, to determine the mechanisms underlying these relationships and changes.

To date, neuroimaging studies have implicated several brain areas as playing a role in attentional processing such as the cingulate cortex (CC), prefrontal cortex (PFC), and hippocampus^32–34^. Interestingly, the CC, PFC, and hippocampus have also been demonstrated to exhibit MMT elicited neuroplastic changes^35–37^. For example, while previous functional magnetic resonance imaging (fMRI) studies have attributed the anterior cingulate cortex (ACC) to the processing of executive control^38^, several studies were also able to attribute greater ACC activation in experienced meditators^39,40^ in addition to observing an increase in ACC activation as a direct result of MMT^35^. Interestingly, improvements in executive control have also been observed following short MMT interventions, which comprised of 20-min practice sessions per day for 3 to 5 days^41,42^. In fact, a recent short-MMT study (i.e., a 4-day Templestay project, which consists in a four-day intensive mindfulness retreat based on Korean Buddhism) on naïve meditators was able to demonstrate a direct association between an improvement in executive control and increased activation in both the right ACC and the right dorsolateral prefrontal cortex (DLPFC) using the ANT^43^. The finding observed in the DLPFC is also supported by another study, in which an increased activation in the right DLPFC and decreased activation in the rostral prefrontal cortex (PFC) was observed in naïve-meditators after an 8-week, focused attention, MMT program^44^. The PFC, an essential brain area for executive functions including working memory, rule learning, planning, attention, and motivation^45^, has therefore been demonstrated to be susceptible to exhibiting neuroplastic changes upon completing MMT. Moreover, several resting-state neuroimaging studies were able to determine that MMT increases the functional connectivity between the posterior cingulate cortex (PCC), dorsal ACC, and DLPFC^46–49^.

Additionally, studies observing microstructural connectivity (i.e., white matter tracts) via diffusion tensor imaging (DTI) as well as regional grey matter volume via voxel-based morphometry were able to demonstrate neuroplastic changes as a consequence of engaging in MMT. For example, a recent MMT study found that naïve mediators, upon completing the eight-week mindfulness-based stress reduction (MBSR) training developed by Jon Kabat-Zinn^1^, exhibited increased microstructural connectivity in the superior longitudinal fasciculus (SLF)^47^, which is a white matter tract connecting the PCC and the DLPFC^50^. Other studies, in addition to observing changes in the SLF, observed increased connectivity between the corpus callosum and corona radiata, which are tracts connecting the ACC with diverse brain areas^51,52^. Furthermore, studies investigating gray matter structure observed volumetric changes in brain areas that play an important role in attention such as the PCC and hippocampus^53,54^.

Interestingly, the hippocampus is another brain area particularly susceptible to both stress and MMT-induced connectivity- and volumetric changes^55–57^. In fact, prolonged, elevated, cortisol levels (i.e., a stress hormone) has been associated with lower hippocampal volume^58,59^. Chronic exposure to cortisol is known to cause neuronal damage particularly in the hippocampus; it also reduces synaptic connections and hippocampal neurogenesis, being presumably reflected in a decrease of hippocampal volume^60,61^. On the other hand, studies have observed larger hippocampal volumes in long-term meditators^62^. Moreover, a study investigating the role of the hippocampus in attention in children and adolescents found a significant correlation between increased hippocampal volume and improved auditory attention^63^. However, the association between the hippocampus, improved attention, and decreased stress levels as a result of MMT remains unknown.

It is important to note, that many of the previously mentioned MMT studies were provided with an in-person MMT, and not a web-based MMT as in the present study. In-person MMT tend to be very expensive, require a specific time commitment, and have a limited group capacity. Web-based MMT on the contrary offer a very flexible schedule, sessions that can be accessed multiple-times, a cost-effective training, and an unlimited number of participants can be included in the training. Due to technological development, and events such as COVID-19, research on web-based MMT is rising, and while initial research on web-based MMT demonstrates improved self-compassion, perceived stress, cognitive skills, mindfulness, and reduced anxiety and depression symptoms^64–67^, there is a need of stronger and robust evidence, as the majority of these studies did not use an active control group to proof the efficacy of web-based MMT. Moreover, a recent review of MMT studies investigating effects on attention also highlighted the need of implementing active control groups to reliable study the effects of MMT, specifically, on attention^68^. Therefore, in the present work we used a study design that included an active control group to elucidate whether a web-based MMT can effectively reduce stress and anxiety levels, improve attention, states of mind, and physical well-being. Also, to the extent of our knowledge, this is the first neuroimaging study using a web-based MMT to identify attentional improvement mechanisms on the neuronal level. If successful, this could provide a larger portion of the population with a validated tool to improve their overall mental health and physical well-being.

The present study, therefore, sought to investigate the effects of a web-based MMT on attention and its association with changes in stress, anxiety, and flow state on both the behavioral and neuronal levels. To achieve this aim, this study utilized state-of-the-art magnetic resonance imaging (MRI) methods and robust statistical analyses procedures. The study had an active control group, pre- and post-intervention measurements (i.e., longitudinal study), and was registered as a clinical trial.

We hypothesized that the web-based MMT would elicit a reduction in stress and anxiety levels in addition to improvements in attention, perceived mindfulness, physical well-being, and flow experience. We expected to observe these behavioral changes accompanied by changes in brain function and structure in the form of increased activation in areas of the CC and PFC during the ANT and increased fractional anisotropy (FA) in white matter tracts connecting significantly activated areas seen in the ANT as a result of the web-based MMT.

## Methods

### Participants

Participants were mainly recruited at Klinikum rechts der Isar, Munich, Bayern, Germany via flyer distributions, online advertisements, and word-of-mouth. The study was advertised as a health-improvement program to ensure that participants were not aware of the mindfulness intervention during the recruitment or start of the experiment. Participants were recruited based on the following inclusion criteria: (1) No prior or current psychiatric or neurological conditions; screened for using the Mini-International Neuropsychiatric Interview (M.I.N.I)^69^, (2) no psychotropic drugs use (including Cannabis), (3) meditation-naïve (i.e., participants should not have had more than three meditation sessions in the past year or more than ten meditation sessions over the course of their life), (4) right-handedness, (5) proficiency in the German language, and (6) age between 18 and 65 years. Additional exclusion criteria for the MRI sample were: (1) Pregnancy, (2) non-removable piercings, (3) tattoos on the head or neck, (4) metal parts or implants in the body, and (5) claustrophobia.

**Figure 1:**
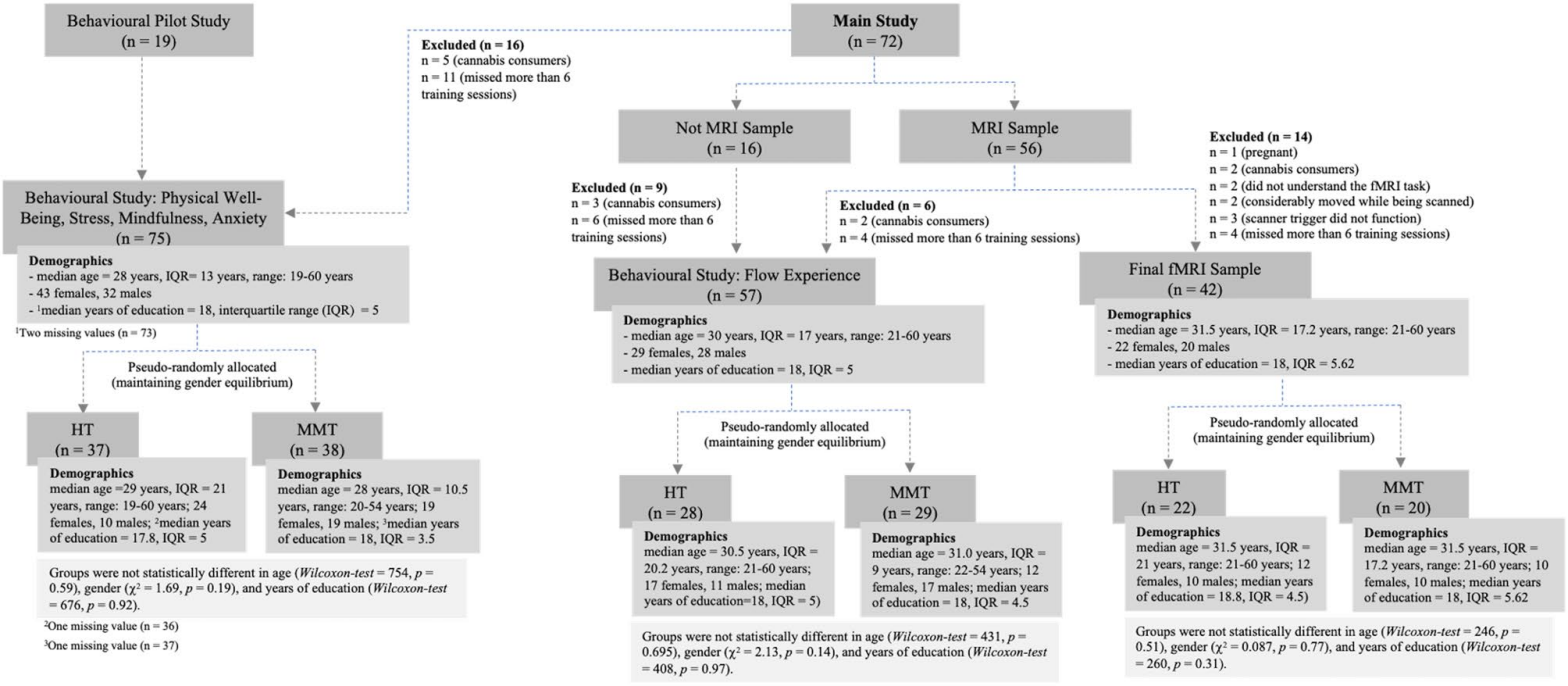
Demographics and statistics of study participants.

In a preliminary stage of the experiment, 19 participants were recruited for a pilot study to evaluate the effects of the web-based MMT on the behavioral level. Here, the effectiveness of the MMT on psychological outcomes, and cognitive function, specifically attention was observed. Following the completion of the pilot study, 72 additional participants were recruited to participate in the main, neuroimaging, study. For the main behavioural study (i.e., measurement of psychological outcomes) a total of 75 participants (43 females and 32 males; $\widetilde{age:}$ 24 years, IQR (interquartile range): 13 years; $\widetilde{YOE}$ (years of education): 18 years, IQR: 5 years) were included. For the main study, flow experience was included as a psychological variable of interest. As this measurement was not added in the behavioural pilot, we were able to include only 57 participants (29 females and 28 males; $\widetilde{age:}$ 30 years, IQR: 17 years; $\widetilde{YOE}$:18 years, IQR: 5 years) for the flow experience sample. Finally, for the main MRI study only 42 (22 females and 20 males; $\widetilde{age:}$ 31.5 years, IQR: 17.2 years; $\widetilde{YOE}$:18 years, IQR: 5.62 years) out of 72 recruited participants survived the inclusion criteria. HT and MMT groups were not statistically different in age, gender, and YOE. More detail can be seen in Fig. 1 where a flowchart containing the demographics of the participants included in the pilot study, behavioral study, and fMRI study is depicted. All participants received monetary compensation for their participation.

### Procedure

The study was registered as a clinical trial using the ISRCTN registry: trial ID ISRCTN95197731. Written informed consent was obtained from all participants and the study was approved by the ethics committee of Klinikum rechts der Isar, Technical University of Munich. Subjects were screened and scanned no more than 1 week prior to their first training session. All participants (in both the pilot and main study) completed a series of standardized questionnaires prior to- and upon completing the MMT or HT. The questionnaires were selected to assess stress levels (Perceived Stress Scale, PSS^70^), perceived mindfulness (Mindful Attention Awareness Scale –German version, MAAS^71,72^), anxiety levels (State and Trait Anxiety, STAI^73^), physical well-being (Fragebogen zur Erfassung des körperlichen Wohlbefindens, FEW-16^74^), and flow experience (Flow Short Scale, FSS^75^).

Participants in the imaging component of the main study were scanned on a 3T Philips Ingenia MR-Scanner (Philips Healthcare, Best, The Netherlands). During the scanning session, a series of neuroimaging sequences were acquired. During the fMRI sequence, participants completed an adapted, event-related, ANT to evaluate attention and its three attentional domains including the alerting, orienting, and executive control networks^76^. Following the pre-training assessment and scanning (TP1), participants were assigned to either the MMT or HT in a pseudo-randomized and single-blinded (subjects-only) manner. Once participants completed their assigned 31-day training program, they were asked to complete a post-training assessment (TP2) within two weeks following the end of their program. This post-training assessment consisted of the same questionnaires applied at TP1, and a second scanning session using the same protocol as TP1. During the TP2 assessment, participants were additionally asked about their compliance with the training program schedule. All participants (in both the MMT and HT groups) needed to complete at least 25 out of the 31 sessions to be included in the study. Enrollment in their respective training was confirmed using the training platform website (https://teachable.com).

### Training

Both the MMT and HT were available online to study participants via the teaching platform www.teachable.com. Participants were given clear instructions on how to create a username to access free of cost their respective training. To ensure that only the content of the two training programs differed from one another, the training programs were structured in an identical manner, by means that both the MMT and the HT each presented a brief, 15-min, video every three days (starting on the first training day), followed by two days of 15-min podcasts or audio recordings. This pattern repeated for the duration of the 31-day course. Videos and audios were supported with written text providing participants with the most important information from the training session of the day. A detailed overview on the structure and content of both the MMT and HT can be found in the supplemental materials (S1).

### Mindfulness meditation training

The MMT utilized in this study was based on the MBSR program and developed free of charge in close cooperation with Dr. Britta Hölzel (BKH), an MBSR instructor and mindfulness researcher, and contains guided mindfulness meditations and exercises, in addition to theoretical concepts and explanations provided in German by BKH. More specifically, theoretical topics included mindfulness research, mind wandering, body awareness, stress physiology, dealing mindfully with pain and difficult emotions, loving kindness, self-perception, connectedness, and others. Guided meditations instructed participants to focus on various objects of attention, such as the breath, body sensations, emotions, thoughts, and walking, in addition to encountering these experiences without judgment, with acceptance, and with kindness. Loving kindness and open monitoring practice were also included.

### Health training

The HT was developed as an active control training program that closely resembled the structure of the MMT but instead gave information on topics pertaining to everyday health. It is important to note that the HT did not contain any information or active training related to mindfulness meditation or meditation in general. Instead, the HT provided participants with health-related topics such as sleep, burn-out, aging, pain, and nutrition.

### Attentional network test

**Figure 2:**
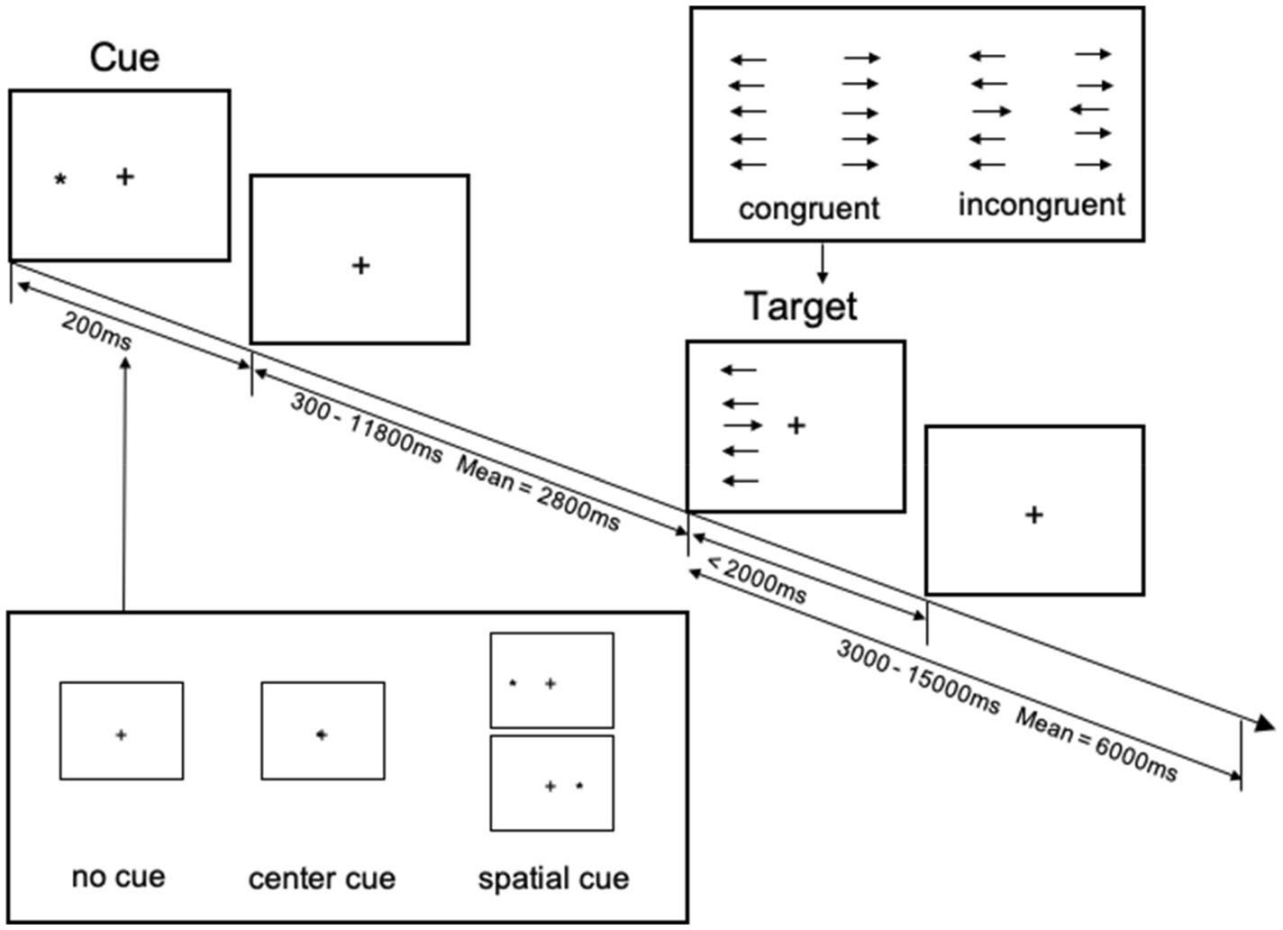
This figure depicts a simplified version of the ANT. Arrows were presented in a column on either the right or left side of the screen. Here, it is important to note that the arrangement of the arrows deviated from the traditional ANT paradigm, such that the arrows were presented in a vertical column, as opposed to in a horizonal row. This was done as in-house pilot experiments indicated a greater effect in the orienting network of attention when the arrows were presented in the vertical arrangement. This figure was adapted from^76^.

The ANT is a paradigm that assesses different forms of attention (i.e., alerting, orienting, and executive control). An adapted version of the ANT was used during the event-related fMRI sequence to observe the neuronal activation patterns of these attentional networks. The ANT consisted of three cue conditions (no cue, center cue, spatial cue) and two target conditions (congruent target, and incongruent target). The no cue condition (i.e., the baseline condition) consisted of a fixation cross presented in the center of the screen. The center cue condition consisted of a fixation cross with an asterisk overlaid in the center of the screen; the function of the center cue condition was to alert participants about the onset of the upcoming target stimulus. The spatial cue condition consisted of an asterisk displayed on either the left or right side of the side of the screen; the function of the spatial cue condition was to orient the attention of the participant to the direction of the upcoming target. Each cue condition had a duration of 200 ms. The cue condition was then followed by one of two target conditions (the congruent or incongruent conditions). The target conditions consisted of a column with 5 horizontal arrows pointing either leftward or rightward. The objective of this condition was for the participants to determine the direction of the center arrow. In congruent conditions, all arrows pointed in the same direction, whereas in the incongruent condition, the center arrow pointed in the opposite direction, thereby introducing a response conflict. Reaction times (RTs) to target conditions were measured. Participants were instructed to use either the index- or middle finger of their right hand to indicate via a button-press whether the center arrow was pointing to the left or right, respectively. The experiment consisted of two runs each comprising of 36 trials. A full trial is depicted in Fig. 2. Congruent and incongruent conditions were counterbalanced and randomly generated. The ANT paradigm for the fMRI session was programmed and presented to the participants using the Presentation^®^ software (Version 20.1, Neurobehavioral Systems, Inc., Berkeley, CA, United States, www.neurobs.com).

### Acquisition of MRI data

All MR imaging was performed on a 3T Philips Scanner, equipped with a 32-channel head coil at Klinikum Rechts der Isar in Munich, Germany. Whole brain functional scans, with 63 anterior commissure—posterior commissure (AC-PC) axial slices (0.2 mm interslice gap) were acquired using a T2*-weighted 2D single shot gradient-echo echo-planar imaging (GE-EPI) sequence, multi-band (MB) Factor = 3, echo time (TE) = 33 ms, repetition time (TR) = 1550 ms, flip angle = 70°, field of view (FOV) = 192 × 192 × 138.4 mm^3^, and an 8 mm^3^ isotropic voxel resolution. The total ANT-fMRI acquisition time was 13 min. To estimate the corresponding field maps to correct for EPI-distortions^77^, two T2*-weighted images were acquired using a gradient echo sequence with two different TE lengths (long TE = 10.54 ms, short TE = 6.0 ms); here, the FOV, spatial resolution, and number of slices were the same as for the functional scans. Structural scans were acquired, with 230 AC-PC axial slices (0.7 mm interslice gap) using a T1-weighted MPRAGE sequence with TE = 5.2 ms, TR = 11 ms, flip angle = 8°, FOV = 256 × 240 × 161 mm^3^, and a voxel resolution of 0.7 ×  0.7 × 0.7 mm^3^. For DTI, diffusion-weighted images (DWI) were acquired with a TR = 5643 ms, TE = 96 ms, FOV = 224 × 256 × 132 mm^3^, and an isotropic voxel resolution of 2 mm. Diffusion-sensitizing gradient echo encoding was applied in 64 directions using a diffusion-weighting factor (*b*) of 1400 s/mm^2^. The total DWI acquisition time was 8 min. The following sequences were additionally acquired during the imaging protocol: Fluid-attenuated inversion recovery (FLAIR, for clinical purposes), resting-state fMRI, and pseudo-continuous arterial spin labeling (pCASL, to evaluate MMT effects on cerebral perfusion). Results of the resting-state fMRI and perfusion study are reported in^49^ and^78^, respectively .

### Analysis of DTI data

The DTI data was first denoised using the *dwidenoise* algorithm from MRtrx3^79^. The ExploreDTI software^80^ was used to conduct signal drift correction, Gibbs ringing correction, Venetian Blinds correction, motion, and EPI/eddy current distortion corrections. The preprocessed images were subsequently fitted to the constrained spherical deconvolution (CSD) model at each voxel^81,82^. FA maps of the white matter tracts of interest were calculated and extracted using the automated/atlas-based region of interest (ROI) analysis of Explore DTI. The tracts of interest were selected based on the significant activations observed in the group-by-time interaction in the fMRI data from the ANT. These tracts included the SLF and the right uncinate fasciculus (rUNC). The atlas “JHU ICBM-DTI-81 White-Matter Labels”^83^ implemented in the ExploreDTI software was used to extract the FA values of the tracts of interest. To observe the group-by-time interaction effects, a two-way mixed ANOVA (with group as the between-subjects factor and time as the within-subjects factor) was conducted on the FA values of the SLF and rUNC. Statistical analyses were conducted using R^84^. Multiple comparison corrections were applied using the Bonferroni method^85^. Statistically significant changes in FA were visualized using raincloud plots^86^. To visualize an example of the white matter tracts of interest, CSD-tractography in manually selected ROIs was done on a control and an experimental participant using Explore DTI.

### Analysis of event-related functional MRI data

Preprocessing and voxel-based analysis of the functional images were conducted using statistical parametric mapping (SPM12, The Wellcome Centre for Human Neuroimaging, London, UK). Participants with a framewise displacement (FD_mean_ > 0.25) were excluded^87,88^. Our pipeline to preprocess the data was as follows: Realignment to the mean functional imaging and unwarping of fMRI time-series, co-registration of anatomical MRI to mean functional image, segmentation of anatomical images, creation of a group-specific DARTEL template^89^ for normalization purposes, normalization to MNI space, and smoothing with a 4 mm FWHM Gaussian Kernel. Slice time correction was not performed as a multiband sequence was used to acquire the data and the TR used was less than 2 s, making the acquisition robust enough to avoid slice timing problems^90^.

Based on the general linear model, a canonical hemodynamic response function was convolved on the event onset-times within the time series to create a statistical model of the ANT for each subject. Design matrices of the first level analysis consisted of five regressors: no cue (i.e., fixation cross), center cue, and spatial cue, in addition to the congruent- and incongruent targets. Six additional nuisance regressors pertaining to movement translations (x,y,z) and rotations (rx, ry, rz) were added to the design matrix. The second level analysis was performed using a two-way repeated measures full factorial ANOVA on the contrasts of interest: alerting network (center cue—no cue), orienting network (spatial cue—center cue), and executive attention network (incongruent target—congruent target). The height (intensity) threshold was set to uncorrected *p* = 0.05. Multiple comparison correction at *p* < 0.05 was determined by a Monte Carlo simulation yielding a cluster size threshold of 350 voxels (2 × 2 × 2 mm^3^). The parameters of the simulation were as follows: SPM volume in voxels (x = 64, y = 77, z = 50), local *p* = 0.05, one tail, global *p* = 0.05, fwhm = 2 voxels, number of iterations = 1500, *t*-distributed, *df* = 80, number of maps = 3 (https://github.com/mbrown/fmrimontecluster/blob/master/fMRIMonteCluster.m by Grown, M. R. G. 2013). Parameter estimates of the activated clusters were obtained using MarsBaR^91^. 3D visualizations of fMRI images were created following the Madan^92^ Guide.

### Behavioral data analyses

Training effects on the attentional networks were assessed using three three-way mixed ANOVAs on the RTs of the ANT, as seen in^43^, with time (TP1 and TP2) and network conditions as the within-subject factors (i.e., no cue and center cue for the ANOVA of the alerting network condition; center cue and spatial cue for the ANOVA of the orienting network condition; congruent and incongruent target for the ANOVA of the executive network condition), and group (MMT and HT) as the between-subject factor. Effects on anxiety, stress levels, mindfulness, flow experience, and physical well-being were assessed by two-way mixed ANOVAs. Data distributions of the behavioral questionnaires are visualized in raincloud plots^86^. Effect sizes were calculated using Cohen’s *d* (Eq. (1)). Statistical analyses were conducted using R^84^.

1 $$Cohen^{\prime}s\, d = \frac{\mu }{{\sigma \sqrt[2]{1 - r}}},\, where \, \mu = \overline{{RT_{TP1} - RT_{TP2} }} \;\;\;  RT = Reaction\,Time,\,TP = Time \,Point$$

### Correlation between brain activation, ANT, and psychological outcomes

Based on our research interest and on the statistically significant behavioral results (i.e., the observed reduced stress and anxiety levels, and increased flow state after the MMT intervention), Pearson’s correlations (*r*) between (a) PSS and ANT-Reaction times, (b) PSS and brain activations, (c) ANT alerting network effect (center cue RT—no cue RT) and brain activations, (d) trait anxiety and brain activations, and (e) flow experience and brain activations were assessed. Pearson’s correlations were corrected for multiple comparisons using the Holm method^93^. Python programming language (Python Software Foundation, https://www.python.org/) was used to perform correlation analysis.

### Ethics approval

The study protocol was approved by the ethics committee of Klinikum rechts der Isar, Technical University of Munich.

### Informed consent

Written informed consent was obtained from all individual participants in the study.

## Results

### Standardized questionnaires results

**Figure 3:**
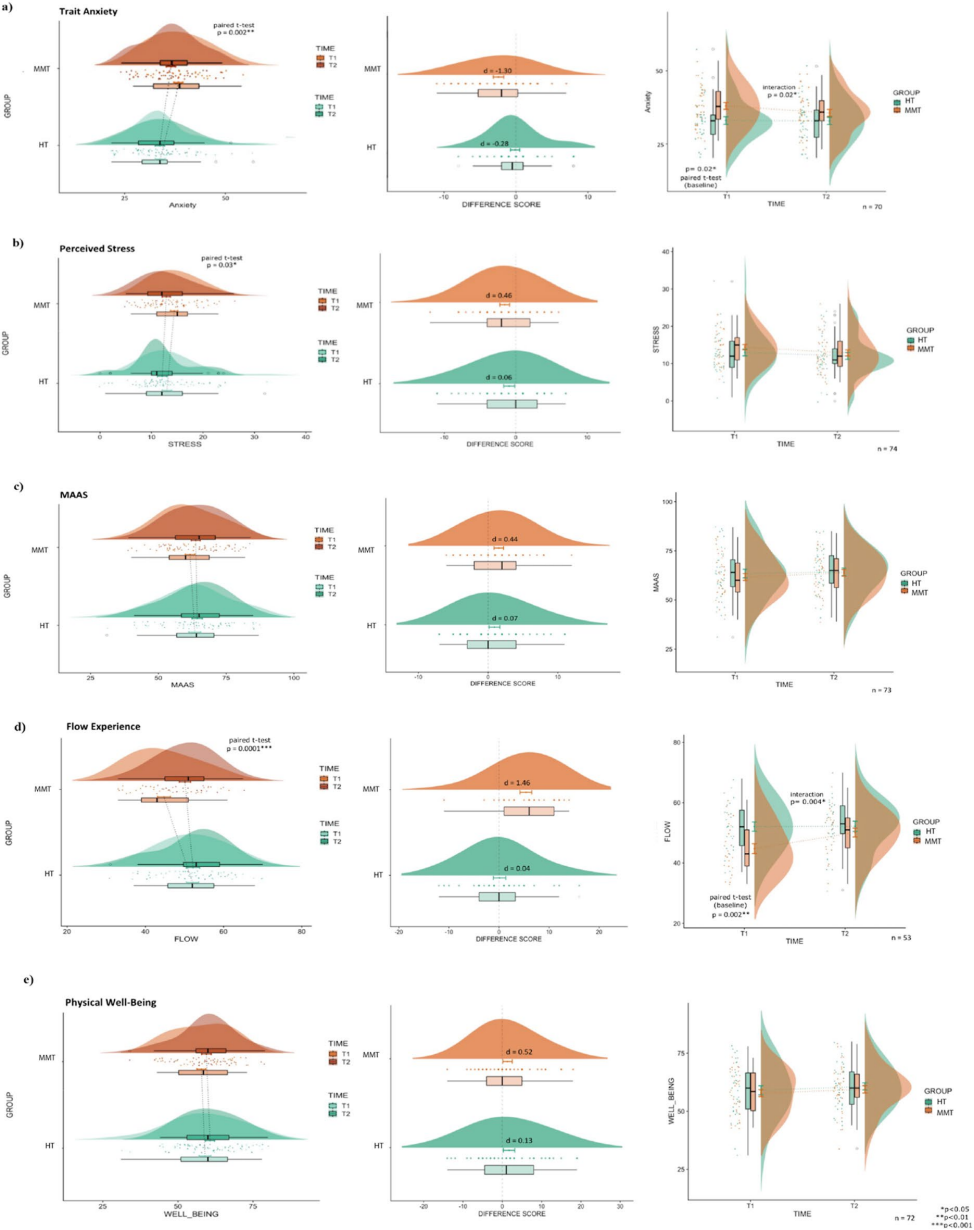
Results of trait anxiety, perceived stress, MAAS (i.e., mindfulness), flow, and physical well-being questionnaires are presented in Rain-Cloud Plots. Significant* p*-values (*p*), Cohen’s *d* effect sizes (*d*), and sample size (*n*) are shown.

When conducting a two-way mixed ANOVA on trait anxiety we were able to observe a significant group-by-time interaction (*F*(1,68) = 5.52, *p* = 0.02). Post-hoc paired *t*-tests confirmed a significant decrease in trait anxiety (*t*(35) = 3.29, *p* = 0.002) with a large effect size (Cohen’s *d* = − 1.30) in the MMT group; this significant decrease was not observed in the HT group (*t*(34) = 0.80, *p* = 0.43, Cohen’s* d* = − 0.28). It is important to note that the baseline value for trait anxiety was statistically different between the MMT and HT groups (*t*(73) = 2.33, *p* = 0.02) (Fig. 3a).

While the perceived stress questionnaires did not yield significant group-by-time interaction when conducting the two-way mixed ANOVA, we were, however, able to observe a significant decrease in stress levels in the MMT group at the second time point (TP2) (*t*(36) = 2.25, *p* = 0.03, Cohen’s* d* = 0.46), whereas no significant decrease in stress levels were observed in the HT group at TP2 (*t*(36) = 1.20, *p* = 0.24, Cohen’s* d* = 0.06) (Fig. 3b).

Additional behavioral measures of interest were mindfulness and flow. The two-way mixed ANOVA of the MAAS did not yield a significant interaction (*F*(1,71) = 1.120, *p* = 0.3) (Fig. 3c). A significant group-by-time interaction was, however, observed for the two-way mixed ANOVA of flow experience (*F*(1,51) = 9.254, *p* = 0.004). Post-hoc paired t-tests demonstrated a significant increase in flow experience for the MMT group (*t*(24) = − 4.56, *p* = 0.0001) with a large effect size (Cohen’s* d* = 1.46), which was not observed in the HT group (*t*(27) = − 0.09, *p* = 0.93, Cohen’s* d* = 0.04). It is important to note that the baseline value for flow experience was statistically different between the MMT and HT groups (*t*(51) = − 3.24 , *p* = 0.002) (Fig. 3d).

For our final measurement of interest, physical well-being, a moderate effect size (Cohen’s* d* = 0.52) was observed indicating an improvement in perceived physical well-being in the MMT group; however, this result was not statistically significant. No effect was observed in the HT group (Cohen’s* d* = 0.13) (Fig. 3e).

### ANT reaction times results

**Figure 4:**
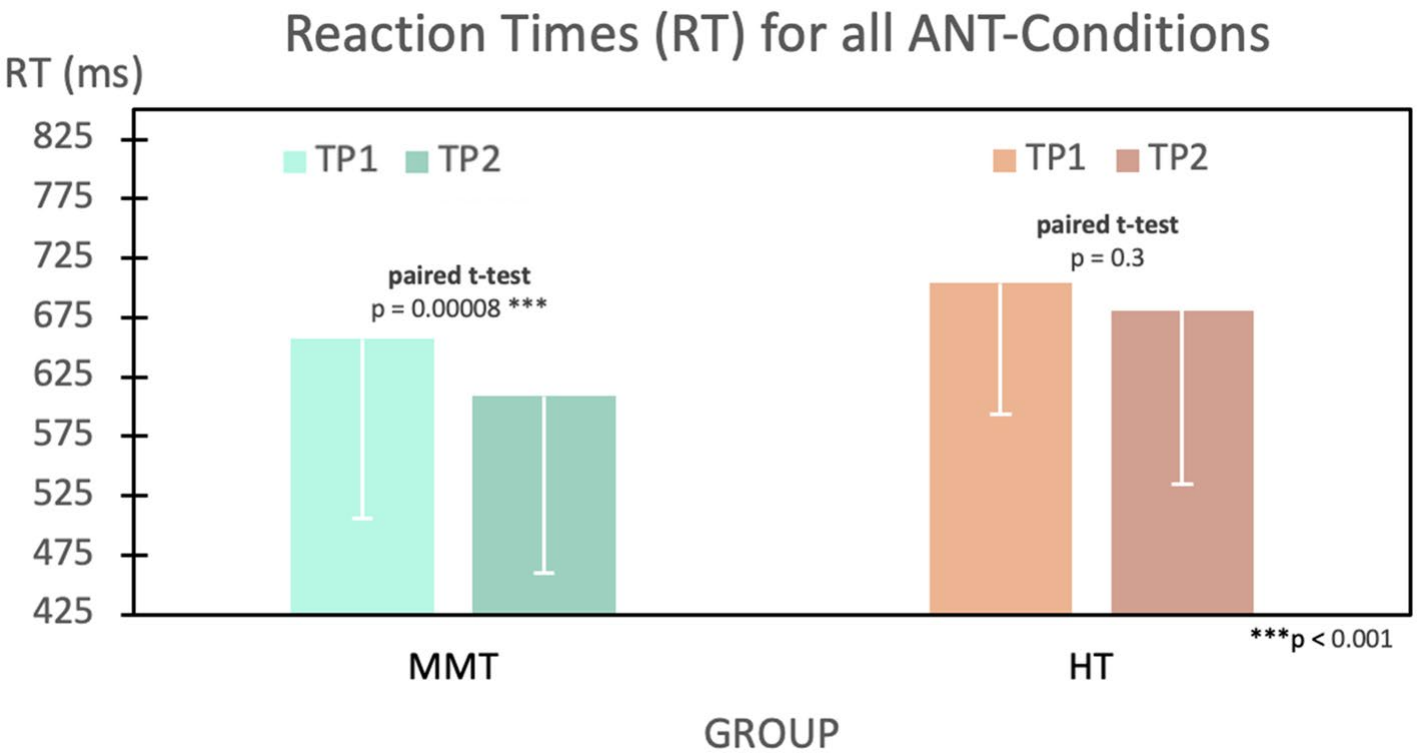
Mean reaction times (mean ± SD) in ms of the MMT and HT groups before and after the intervention over all conditions.

Interestingly, the mean overall RT significantly improved by ~ 48 ms (*t*(1,19) = 5.07, *p* = 0.00008) in the MMT group at TP2, whereas the mean RT for the HT only decreased by ~ 23 ms, which was not statistically significant (*t*(1,22) = 1.10, *p* = 0.285) (Fig. 4). These results indicate that there was more than a two-fold RT improvement in the MMT group compared to the HT group. Despite these striking results for overall RT, this effect was not specific when considering the individual RTs of the three attentional conditions (i.e., the alerting-, orienting-, and executive- conditions) and conducting a three-way mixed ANOVA for each of them, with group as a between-subjects factor, time and condition as within-subject factors, yielded no significant group-by-time-by-condition interaction across the different conditions.

In the following, all significant interactions are reported for a complete overview of the finding. A significant two-way interaction (*F*(1,39) = 6.811, *p* = 0.01) between group (i.e., MMT and HT) and condition (i.e., center cue and spatial condition) for the orienting condition of attention was observed. The post-hoc two-way ANOVA analysis revealed a significant main effect of group in the spatial cue condition (*F*(1,163) = 4.84, *p* = 0.03), indicating that the MMT group demonstrated faster spatial cue RT at baseline and at TP2. As expected, a main effect of condition for alerting (no cue and center cue) (*F*(1,39) = 11.097, *p* = 0.002) and executive attention (incongruent and congruent) (*F*(1,40) = 34.39, *p* < 0.001) was observed, indicating that, when the condition was more cognitively effortful (i.e., complex) the reaction time increased. Furthermore, and not surprisingly, main effects of time were observed for each attentional condition (*F*(1,39)_Alerting_ = 16.469, *p* < 0.001; *F*(1,39)_Orienting_ = 21.16, *p* < 0.001; *F*(1,40)_Executive_ = 15.53, *p* < 0.001), indicating practice effects from the first to second testing timepoints.

As seen by the effect sizes for the change in RT in each group (Table 1), the effects of the intervention were not specific to a particular attentional condition, indicating that, the RTs significantly improved for the MMT group in all attentional conditions, and did not have an effect on any particular attentional network.

**Table 1:** ANT effect sizes for the difference in RT between the two timepoints for each group.

Cue	Target	Cohen’s *d*	
		HT	MMTs
No	Congruent	0.24	2.3
	Incongruent	0.40	3.2
Center	Congruent	0.46	3.1
	Incongruent	0.26	3.3
Spatial	Congruent	0.78	1.4
	Incongruent	0.43	3.9

The Cohen’s *d* (*d*) effect size scale is: negligible effect (*d* < 0.2), small effect (0.2 ≤ *d* < 0.5), moderate effect (0.5 ≤ *d* < 0.8), and large effect (*d* ≥ 0.8)^94,95^.

### fMRI results

**Figure 5:**
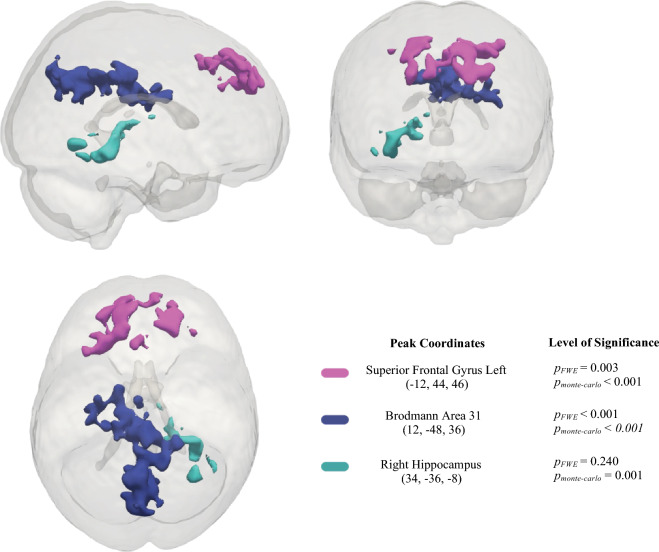
Neuroimaging results in MNI space of the two-way repeated measures, full factorial, ANOVA for the alerting network of attention. This figure shows the significant brain activations when comparing the MMT and HT groups.

**Figure 6:**
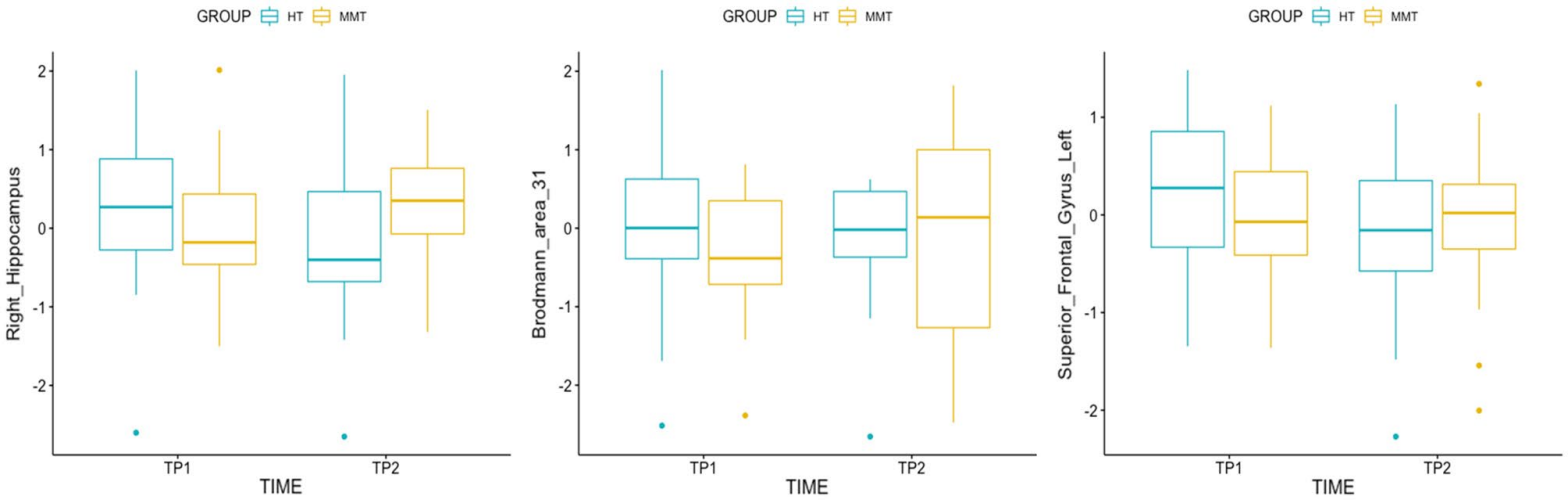
Parameter estimate boxplots illustrating the directionalities of activation in the right hippocampus, Brodmann area 31, and left superior frontal gyrus in both the MMT and HT at each time point.

To determine whether there was a significant change in brain activation during the ANT at TP2 as a result of the MMT, a whole brain, two-way, repeated measures full factorial ANOVA was conducted (Table 2). This analysis demonstrated a significant group-by-time interaction during the alerting condition in which an increase in activation in the left superior frontal gyrus (SFG; *p*_*FWE*_ = 0.003, *p*_*monte-carlo*_ < 0.001), Brodmann area 31 (BA 31; *p*_*FWE*_ < 0.001, *p*_*monte-carlo*_ < 0.001), and the right hippocampus (p_FWE_ = 0.240, *p*_*monte-carlo*_ = 0.001) in the MMT group (in comparison to the HT group) was observed. Figure 5 illustrates the significantly activated clusters from the group-by-time interaction, and Fig. 6 depicts the change in parameter estimates (β values) of the brain areas exhibiting increased activation in the MMT group via boxplot visualizations. Interestingly, we were also able to observe decreased activation (also visualized in the boxplots of the parameter estimates) in the HT group. The other attentional conditions (i.e., orienting, and executive attention) did not yield significant group-by-time interaction results.

**Table 2:** Regions showing a significant group-by-time interaction for the alerting network of the ANT (no cue—center cue).

Region	MNI coordinates (mm)			Cluster-level		
	x	y	z	*p*_*FWE-corrected*_	*p*_*monte-carlo-corrected*_	*k*^a^
Superior Frontal Gyrus Left	− 12	44	46	0.003	< 0.001	1054
Brodmann Area 31	12	− 48	36	< 0.001	< 0.001	1992
Right Hippocampus	34	− 36	− 8	0.240	0.001	484

^a^*k* = cluster size in voxels.

### DTI results

**Figure 7:**
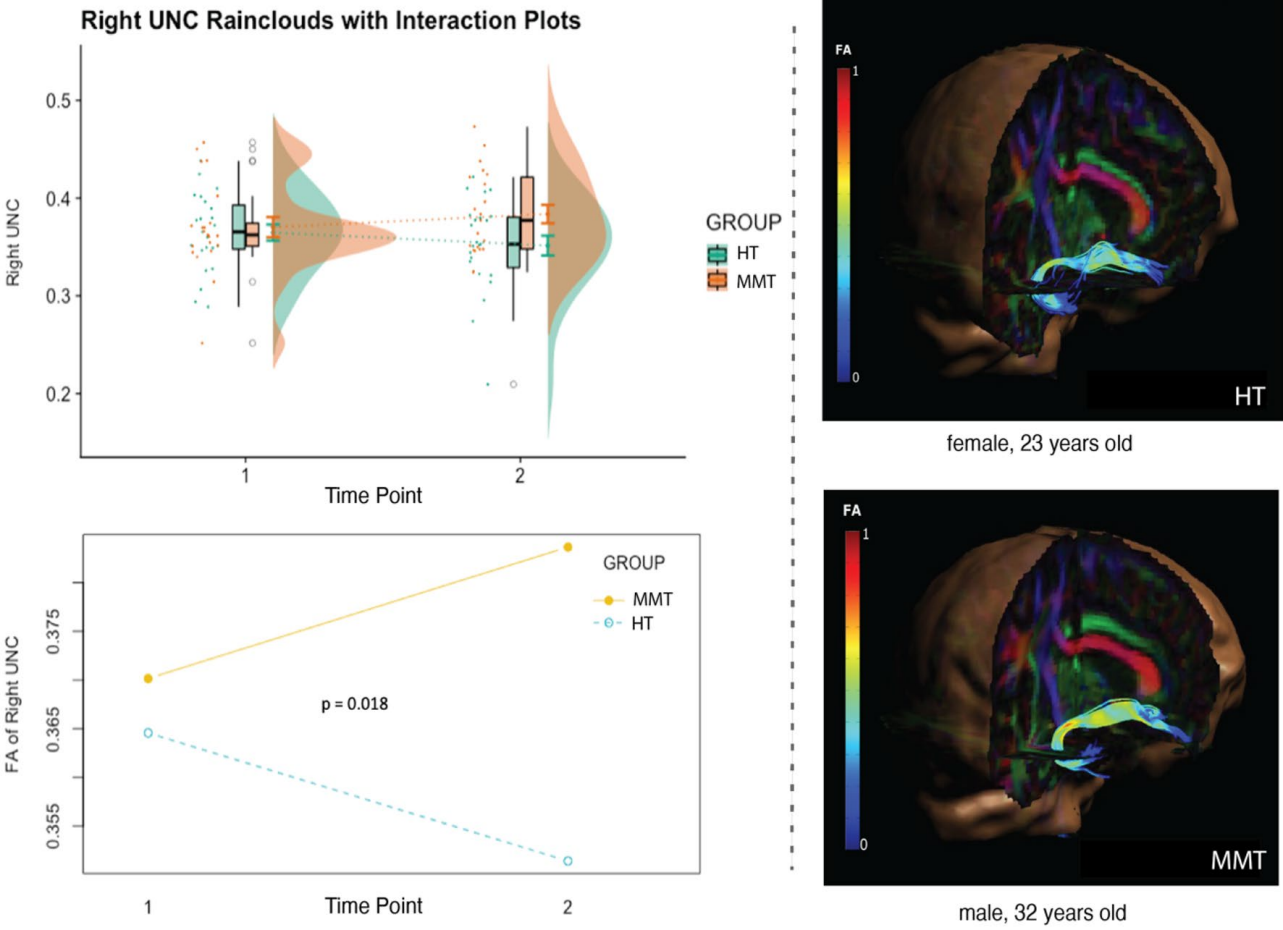
On the left, the change in FA values in the rUNC of the HT and MTT groups is depicted by raincloud and interaction plots. On the right, examples of the tractography of the rUNC of a HT and MMT participant are depicted.

To determine whether the changes observed in brain activation during the ANT were accompanied with microstructural changes in the white matter of the brain, we conducted a DTI analysis on two fiber tracts, namely the right uncinate fasciculus (a tract that connects the PFC with the right hippocampus), and the SLF, a tract that connects the PCC (i.e., BA31) with the PFC. These fiber tracts were selected as they are associated with the significant brain area activations observed during the ANT. The DTI results, through a group-by-time two-way mixed ANOVA, were able to demonstrate a significant training-associated increase in FA in the rUNC (F(1,42) = 6.047, p = 0.018) in the MMT compared to the HT group (Fig. 7); these results survived Bonferroni correction. The group-by-time two-way mixed ANOVA analysis for the DTI results in the SLF yielded no significant interaction.

### Correlations: brain activations in the alerting condition, ANT reaction times, and psychological outcomes

An additional aim of this study was to determine the relationship between the neuroplastic and behavioral (i.e., psychological) changes upon completing the MMT; correlation analyses between the increased training-related brain activations in the ANT during the alerting condition (i.e., SFG, Brodmann Area 31, and right hippocampus) and behavioral measures (i.e., PSS, ANT RTs, trait anxiety, and flow experience) were therefore conducted.

**Figure 8:**
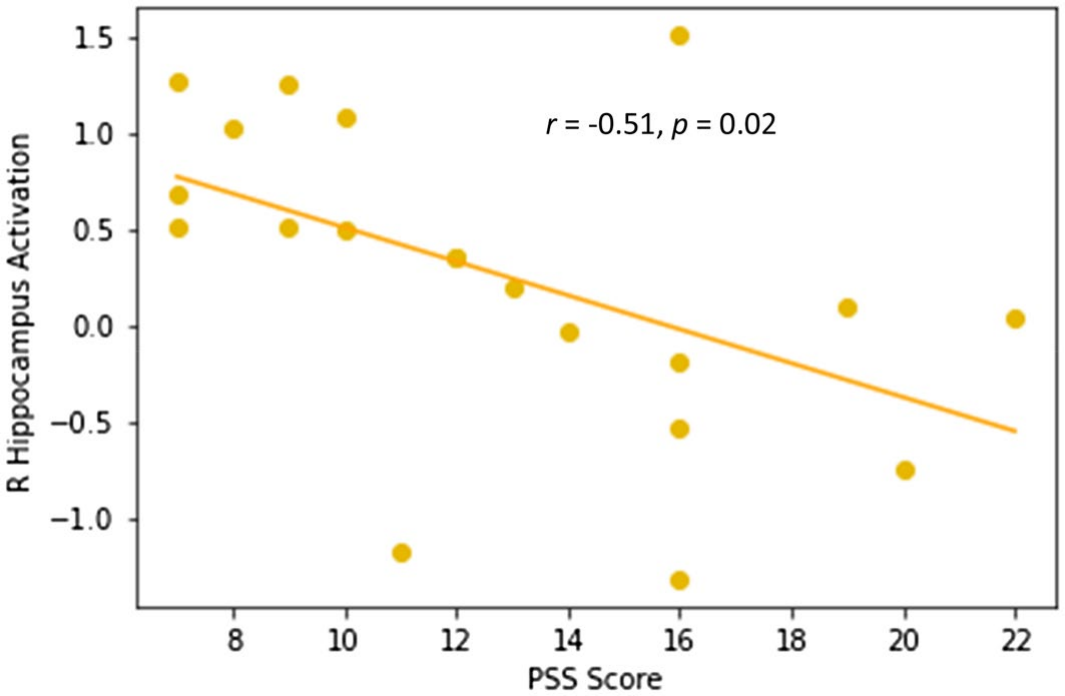
This plot depicts the correlation between the PSS scores and the right hippocampus activation (β values) after completing the MMT (TP2).

The correlation analysis between perceived stress levels and the increased brain activations observed in the ANT yielded a significant negative correlation (*r* = − 0.51, *p* = 0.02, Fig. 8) between PSS scores and the activation in the right hippocampus after MMT that marginally survived the Holm’s correction method for multiple comparisons. This correlation was not observed in the MMT group before the intervention nor in the HT at any of the two time points (Table 3). An additional correlation analysis was conducted to observe whether there was an association between the change in PSS scores and the change in mean ANT reaction time in the MMT group (TP2 vs TP1). This correlation approached significance (*r* = 0.36, *p* = 0.059, one-tailed).

**Table 3:**
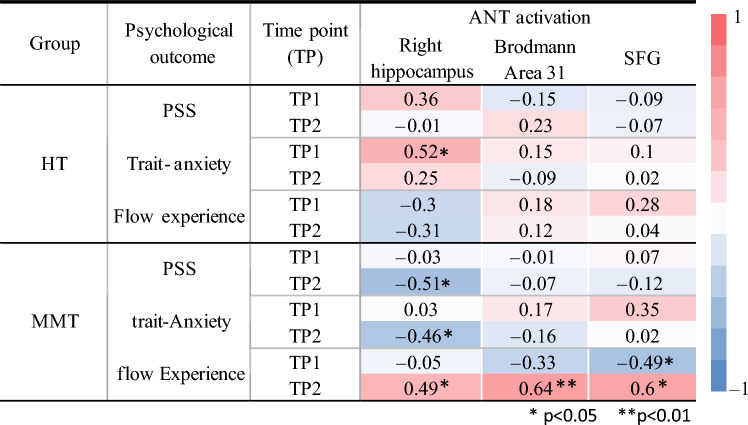
Correlations observed between the psychological outcomes (PSS, trait anxiety, and flow experience) and the training-related increased brain activations (right hippocampus, Brodmann area 31, and SFG) in the HT and MMT group for both TP1 and TP2.

We were also interested in determining whether we could observe a relationship between the alerting effect (no cue—center cue) and the significant brain activations, but no significant correlations were found. However, a trending, positive, correlation (*r* = 0.4) between the change in activation in the SFG and the change in reaction times of alerting cue (i.e., center cue) was observed (*p* = 0.08).

Another correlation of interest within the scope of this study was to determine the relationship between trait anxiety levels and the observed brain activations. The correlation analysis yielded a significant negative correlation (*r* = − 0.46, *p* = 0.04) between the trait anxiety scores and the activation in the right hippocampus in the MMT group at TP2. Interestingly, we were also able to observe a significant positive correlation in the HT group at TP1 (*r* = 0.52, *p* = 0.02). No significant correlations were observed in the MMT at TP1 nor were any correlations observed in the HT group at TP2. However, it is important to note that the positive correlation observed in the HT group at TP1 only marginally survived Holm’s correction for multiple comparisons, while the negative correlation in the MMT group at TP2 did not. No significant correlations were found between trait anxiety and the SFG, nor between trait anxiety scores and the Brodmann Area 31 (Table 3).

Our final correlation of interest was to observe the relationship between flow state and the increased brain activations. The correlation analysis yielded significant, positive, correlations in the MMT group at TP2 between flow experience and the right hippocampus activation (*r* = 0.49, *p* = 0.047), Brodmann area 31 activation (*r* = 0.64, *p* = 0.006), and SFG activation (*r* = 0.6, *p* = 0.01). No significant correlations were observed in the MMT group at TP1 nor in the HT group at TP1 or TP2 (Table 3). All significant correlations observed in relation to flow experience survived the Holm´s multiple comparison correction.

## Discussion

The main aim of the present study was to investigate the effects of a 31-day web-based MMT on attention on both the behavioral and neuronal levels and its relationship with changes in perceived stress, trait anxiety, and flow experience. Through the use of robust analysis methods, the results of this study demonstrated that a brief web-based MMT led to improvements in the behavioral measures of mental health (i.e., reductions in trait anxiety and stress levels), states of mind (i.e., flow experience), attentional performance (i.e., reaction-time improvements), and to significant neuroplastic changes exhibited by an increase in brain activation in the superior frontal gyrus, Brodmann area 31 (i.e., PCC), and right hippocampus during the alerting condition of the attentional task. These results were corroborated by changes observed in the microstructural integrity between the right hippocampus and the superior frontal gyrus (i.e., FA changes in the right uncinate fasciculus). We were able to observe these significant changes on both the behavioral and neuronal levels, and to associate the neuronal changes to our observed improvements in attention as well as to reductions in stress and trait anxiety. Our findings, therefore, contribute to the research conducted on possible interventions for chronic stress, an ever-increasing central health issue.

One of our main findings of interest demonstrated significant MMT-elicited improvements in cognitive and executive functioning, specifically attentional performance. We were able to demonstrate improvements in the overall RT during the ANT and to observe an increase in brain activation in the superior frontal gyrus, PCC, and right hippocampus during the alerting condition of the ANT. These neuronal findings are noteworthy given their association with cognitive and executive functioning seen in previous literature findings. For example, the PCC has been attributed to playing a crucial role in cognitive functioning, specifically with regard to the RT of cognitive performance, and it has been associated with the frontoparietal control network as well as the dorsal attention network, which are both critically involved in visuospatial attention^96–98^. Our results observed in the PCC, therefore, continue to support previous findings reported in the literature.

Interestingly, as the PCC has been demonstrated to be a major node in multiple intrinsic connectivity networks it is directly associated with the hippocampus, ACC, and PFC^96^. Like the PCC, the hippocampus is an important brain region involved in visuospatial attention^99^. More specifically, the dorsal hippocampus plays a role in cognitive functioning, while the ventral hippocampus modulates behavioral adaptation to stress^100^. Our findings observed in the right hippocampus, therefore, support the findings in the literature, while also continuing to emphasize the role the hippocampus plays in cognitive functioning and attention. Similarly, the ACC is a brain region involved in executive functioning and has also been implicated in the effects of mindfulness meditation^39,43,101^. In fact, evidence supports that experienced meditators exhibit increased connectivity between the PCC, ACC, and DLPFC^46^ which are brain areas attributed to cognitive functioning and are also susceptible to the effects of MMT^47,102–104^. In addition, MMT studies conducted on naïve-meditators have also shown an increased resting-state PCC-DLPFC connectivity. The DLPFC, formed by the lateral part of the SFG and middle frontal gyrus^105^, plays an important role in focused attention^106^. Moreover, our observed activations in the SFG, can be linked to previous findings in which the SFG activations were attributed to higher cognitive functioning and, more specifically, to attentional shifting and spatial cognition^107,108^. In fact, patients with attention-deficit/hyperactivity disorder (ADHD) have shown abnormal function and structure in the SFG^109^. Additionally, a study done on an epileptic patient, where activity in the SFG was recorded with subdural electrodes, demonstrated via the Flanker Task (an attention task similar in nature to that of the ANT) that the SFG actively participates in executive control tasks^110^. Therefore, our trending toward significant correlation observed in the MMT group between the increased SFG activation and faster RTs in the alerting condition of the ANT, is in line with this interpretation thus continuing to emphasize the important role MMT can have on improving attention.

Interestingly, we were able to observe both improvements in cognitive functioning as represented in the decrease in RTs during the ANT and a significant group-by-time interaction showing an increase in flow experience in the MMT. These findings are important as it implies that MMT can have an effect on both attentional mechanisms, as well as on the psychological flow state. While the research conducted on flow is still considered to be a relatively new line of research^111^, the neuroimaging results observed in the present study are able to provide initial insight into the association between increased flow and neuroplastic changes through the observed correlation between flow-experience and brain activation in the right hippocampus, PCC, and SFG, during the ANT. This finding, therefore, emphasizes the important relationship between mindfulness, attentional functioning, and flow experience.

Although no significant group-by-time interaction was observed in perceived stress, we were able to demonstrate a statistically significant reduction in the MMT group in stress level (observed via the PSS), not seen in the HT group. It is important to note, that we may not have been able to observe an interaction for the PSS due to the low perceived stress at baseline for both the MMT and HT groups. Therefore, the null result in the group-by-time interaction should be considered with caution, especially when previous research demonstrated MMT’s ability to elicit greater stress reduction in individuals with higher initial stress than participants with lower initial stress levels^112^.

We were also able to demonstrate a negative correlation between hippocampal activation and the PSS score in the MMT group at TP2 (Fig. 8). This finding is significant given the role of the hippocampus in modulating the behavioural adaptation to stress^100^. In fact, previous research has been able to demonstrate a decrease in activation in brain areas associated with the limbic system (i.e., hippocampus, hypothalamus, medio orbitofrontal cortex, and ACC) during acute stress, triggered by an increase of cortisol levels^113^, while also demonstrating that MMT can have an influence on both the functional and structural changes in the hippocampus^53,57,114,115^. Our previous study utilizing voxel-based morphometry to observe MMT-elicited neuroplastic changes, demonstrated greater gray matter concentration in the right hippocampus of meditators^116^, further emphasizing the importance of the increase in hippocampal activity observed in our functional imaging data. We were able to demonstrate an association between trait anxiety, perceived stress, and hippocampal activity during an attention task, and that MMT can have an influence on the white matter microstructure of the brain by means that we observed increased FA in the rUNC. These results indicate that MMT strengthened the connection between the hippocampus and areas of the frontal lobe. In fact, our findings replicate the results of our previous DTI study in which a significant increase in FA in the right UNC following MMT was detected^117^. Other studies have also demonstrated an increase in FA of white matter tracts surrounding the dorsal part of the hippocampus^118^, in addition to associating changes in the left UNC with the experience of silence in a different form of meditation^119^. Our results demonstrating MMT-related activations in the hippocampus and increased FA in the rUNC, taken together with the finding in the literature, suggests a close relationship between structural and functional changes in specific brain regions and, more specifically, that the hippocampus may be a candidate to mediate the relationship between MMT, attention, and stress reduction.

It is, however, important to consider the limitations that accompany the scope of this study. For example, while previous studies demonstrated the effectiveness of a brief MMT on behavioral measures, additional studies were able to demonstrate that psychological outcomes can be positively moderated by the number of MMT hours conducted^2,120^. Therefore, as our MMT was one month shorter in comparison to the standard MBSR program^3^, we expect that by increasing the duration of our web-based MMT, larger effects could be reached. Nevertheless, our findings indicate that engaging in brief web-based MMT can also elicit significant behavioral and neuroplastic changes and is therefore a significant contribution to the literature.

An additional limitation to consider is the small sample size of our neuroimaging data, as it was challenging to find meditation-naïve, MRI suitable participants. And, given the longitudinal nature of this study, we also had an attrition rate of $\sim$ 10%. Moreover, technical issues with the MRI scanner or excessive motion during the scan reduced our sample size. Another disadvantage in our study design was the inability to prove participant compliance in the web-based training programs.

Furthermore, the participant sample in this study exhibited a non-normal distribution for age; this, in turn, may have influenced the observed results, given the differences between the older and younger participants. In fact, previous studies have already reported differences in the impact of MMT between different age groups^121,122^. Nevertheless, the results of this study ultimately contribute to the understanding of the neural mechanisms of MMT on attention, in addition to its impact on psychological outcomes. Future studies should investigate the use of the current web-based mindfulness training in clinical populations in an effort to observe similar results on stress, anxiety, and cognitive capacities.

## Conclusion

This was a complex longitudinal, web-based MMT study involving acquisiton of several MR neuroimaging data, including event-related fMRI and DTI, and assessment of psychological outcomes by means of stadardized questionnaires. These data combined with the inclusion of an active control group successfully and reliably demonstrated that a short web-based mindfulness training has the potential to improve mental health (i.e., by reducing anxiety and perceived stress levels), cognitive performance (i.e., attention mechanisms), and state of mind (i.e., flow experience), which coincide with alterations in underlying brain structure (i.e., increased FA in the rUNC), and brain function (i.e., increased activation in the PCC, right hippocampus, and SFG). These findings provide the scientific community with valuable insight into the relationship between mindfulness training, mental health, cognitive function, and its corresponding neural correlates. More specifically, we showed that the decrease in stress and anxiety in our experimental group is presumably a precursor of improved hippocampus function, reflected in improvements of alerting attention. And finally, this increase in functionality might have caused white matter changes in the rUNC which connects the right hippocampus with frontal areas of the brain known to be involved in attentional processes.

We were also able to demonstrate an important link between the mindfulness meditation training-related increase in flow experience and the SFG, right hippocampus and PCC, which as previously described are important brain areas involved in attentional processes. Furthermore, a recent publication by Xie^123^ was able to demonstrate an association between flow experience, mindfulness, and team-working abilities. The study reported a correlation indicating that productivity was associated with higher mindfulness and flow experience levels. Given these findings, our results may indicate that the web-based MMT used in our study may be suitable for companies and their employers due to its ability to increase flow experience and attention. Previous literature have also suggested a link between mindfulness, flow, and higher workplace performance^124^. Web-based MMTs could, therefore, be beneficial to companies and their employers by increasing workflow experience, and may also help to decrease the stress and anxiety elicited by a stressful workday. As stress and anxiety have been demonstrated to impair cognitive performance and function^125^, MMTs, by decreasing stress and anxiety levels, help to improve overall cognitive function (i.e., attention), thereby improving productivity.

In conclusion, web-based MMT could serve as an important tool to both increase the overall well-being of individuals suffering from stress and anxiety and to improve domains of cognitive function such as attention. Moreover, our web-based MMTs provide a larger portion of the population with a validated tool to improve their overall mental health and physical well-being.

As future directions, we plan to employ the current web-based mindfulness training in clinical populations, such as in obsessive–compulsive disorder (OCD) and ADHD patients, hoping to see similar effects predominantly on stress, anxiety, flow experience, and cognitive capacities. As well as to better understand the neurological mechanisms of action behind these changes, and how these can help in the treatment and prognosis of psychiatric diseases.

### Supplementary Information

Supplementary Information 1.

Supplementary Information 2.

## Data Availability

The datasets generated during and/or analysed during the current study are available in the Open Science Framework (OSF) repository, https://doi.org/10.17605/OSF.IO/NAXC8.
