# Supplementary material for: Effects of web-based mindfulness training on psychological outcomes, attention, and neuroplasticity
Source: Sci Rep. 2023 Dec 19;13:22635. doi: 10.1038/s41598-023-48706-0 (PMC10730881; doi:10.1038/s41598-023-48706-0)
Supplement: Supplementary file 1 — Supplementary Information 1. [file 41598_2023_48706_MOESM1_ESM.pdf]

SUPPLEMENTAL MATERIAL (S1)

		Mindfulness Meditation Training (MMT)		Health Training (HT)
**Session**	**Format**	**Theory**	**Practice**	**Theory**
**1**	Video	Introduction to mindfulness	Mindful breathing A	Sleep
**2**	Audio	*N/A*	Mindful breathing A	Chronic pain
**3**	Audio	*N/A*	Mindful breathing A	Light exposition and health
**4**	Video	Arriving in presence	Mindful breathing B	Sleep disturbances
**5**	Audio	*N/A*	Mindful breathing B	Body memory
**6**	Audio	*N/A*	Mindful breathing B	Migraine
**7**	Video	Arriving in the body	Bodyscan A	Burnout
**8**	Audio	*N/A*	Walking meditation	Nutritional supplements
**9**	Audio	*N/A*	Bodyscan A	Social inequality and health
**10**	Video	Subjectivity of perception	Bodyscan B	Sore muscles / Vegan diet
**11**	Audio	*N/A*	Walking meditation	Time perception
**12**	Audio	*N/A*	Bodyscan B	Gender specific health experience
**13**	Video	Communicating mindfully	Mindful attention to body sensations	Vitamins
**14**	Audio	*N/A*	Mindful attention to body sensations	Health impacts of dieting
**15**	Audio	*N/A*	Mindful attention to body sensations	Aging
**16**	Video	Non-judgement	Mindful attention to body sensations	Sugar
**17**	Audio	*N/A*	Mindful listening	Maintaining a diet
**18**	Audio	*N/A*	Mindful listening	Self-deceit
**19**	Video	Dealing with stress	Mindfully approaching emotions	Raw foods
**20**	Audio	*N/A*	Mindfully approaching emotions	Migration and health

**21**	Audio	*N/A*	Mindfully approaching emotions	Epigenetics
**22**	Video	Turning towards instead of turning away	Turning towards instead of turning away	Sensible footwear
**23**	Audio	*N/A*	Approaching unpleasant feelings	Obsessive-compulsive disorder
**24**	Audio	*N/A*	Awareness of thinking	Self-efficacy
**25**	Video	Positive qualities	Loving kindness	Busting breakfast myths
**26**	Audio	*N/A*	Loving kindness	Cardiovascular diseases
**27**	Audio	*N/A*	Loving kindness	Hypnotherapy
**28**	Video	Decentring	Open monitoring	Staying active in the office
**29**	Audio	*N/A*	Open monitoring	Negative empathy
**30**	Audio	*N/A*	Silent meditation	Pain perception
**31**	Video	Reflecting the course	Silent meditation	Physical activity

N/A: not applicable
