# Supplementary material for: Effects of web-based mindfulness training on psychological outcomes, attention, and neuroplasticity
Source: Sci Rep. 2023 Dec 19;13:22635. doi: 10.1038/s41598-023-48706-0 (PMC10730881; doi:10.1038/s41598-023-48706-0)
Supplement: Supplementary file 2 — Supplementary Information 2. [file 41598_2023_48706_MOESM2_ESM.pdf]

SUPPLEMENTAL MATERIAL (S2)

Abbreviations

ACC	Anterior Cingulate Cortex
AC-PC	Anterior Commissure – Posterior Commisure
ADHD	Attention-Deficit/Hyperactivity Disorder
ANOVA	Analysis of Variance
ANT	Attention Network Test
Brodmann Area 31	BA 31
CC	Cingulate Cortex
COVID-19	Coronavirus Disease of 2019
CSD	Constrained Spherical Deconvolution
DARTEL	Diffeomorphic Anatomical Registration using Exponentiated Lie algebra
df	degrees of freedom
DLPFC	Dorsolateral Prefrontal Cortex
DTI	Diffusion Tensor Imaging
DWI	Diffusion-Weighted Imaging
FA	Fractional Anisotropy
FD	Framewise Displacemente
FEW-16	Fragebogen zur Erfassung des koerperlichen Wohlbefindens
FLAIR	Fluid-Attenuated Inversion Recovery
fMRI	functional Magnetic Resonance Imaging
FOV	Field-of-View
FSS	Flow Short Scale
FWE	Family-Wise-Error
FWHM	Full Width at Half Maximum
fwhm	full width at half maximum in voxels
GE-EPI	Gradient-Echo Echo-Planar-Imaging
HT	Health Training
i.e.	id est (that is)

IQR	Interquartile Range
MAAS	Mindful Attention Awareness Scale
MB	Multi-band
MBSR	Mindfulness-Based Stress Reduction
min	minute
M.I.N.I.	Mini-International Neuropsychiatric Interview
MMT	Mindfulness Meditation Training
MNI	Montreal Neurological Institute
MR	Magnetic Resonance
MRI	Magnetic Resonance Imaging
OCD	Obsessive-Compulsive Disorder
p	p-value
pCASL	pseudo-continuous arterial spin labeling
PCC	Posterior Cingulate Cortex
PFC	Prefrontal Cortex
PSS	Perceived Stress Scale
r	correlation coefficient
ROI	Region of Interest
RT	Reaction Time
rUNC	Right Uncinate Fasciculus
SFG	Superior Frontal Gyrus
SLF	Superior Longitudinal Fasciculus
SPM	Statistical Parametric Mapping
STAI	State and Trait Anxiety Levels
T	Tesla
TP1	pre-training assessment
TP2	post-training assessment
TE	Echo Time
TR	Repetition Time
YOE	Years Of Education

Glossary

INTRODUCTION

Mindfulness meditation training (MMT) is a practice that allows for an increase of awareness to experiences occurring in the present moment such as thoughts, feelings, and body sensations without any judgment.

Posner's attention network model explains attention as a unified process which includes three attentional mechanisms, each has its own functions and networks in the brain.

Alerting network that represents a heightened state of alertness and vigilance. It measures responses to a target that is presented following a cue.

Orienting network involves selecting information from sensory input. It measures responses to a target that is presented in a specific location.

Executive control network involves resolving conflict among responses and error monitoring. It measures responses to a different level of conflicts.

Attention network test (ANT) separately assesses, and measures three attentional mechanisms proposed in Posner's attention network model.

Flow state is an intense state of focus on an activity while an individual may lose track of time with a sense of ease and enjoyment.

Functional magnetic resonance imaging (fMRI) is a neuroimaging technique that correlates brain activity with specific cognitive processes. It measures changes in blood flow and levels of oxygen.

Cingulate cortex (CC) is part of the limbic system and resides on the medial surface of the cerebral cortex. It plays a role in pain responses and emotion.

Posterior cingulate cortex (PCC) is the back part of the cingulate cortex. It is involved in memory functions and regulating the focus of attention.

Anterior cingulate cortex (ACC) is the front part of the cingulate cortex and has connection to the limbic system. It is involved in monitoring and adjusting behavior in response to emotions.

Prefrontal cortex (PFC) is located in the front part of the frontal cortex. It is associated with complex cognitive behaviors like planning and decision making.

Dorsolateral prefrontal cortex (DLPFC) is the posterior and lateral part of the prefrontal cortex. It is associated with executive processings including selective attention and working memory.

Hippocampus is located in the medial part of the temporal lobe. It is involved in learning and memory processes.

Neuroplasticity is the ability of the brain to change and to rewire on neuronal level by experience.

Resting-state neuroimaging is examining the neural activity of the brain while no explicit task is performed.

Functional connectivity is statistically correlated brain signals in time among brain regions.

Diffusion tensor imaging (DTI) is an MRI-based technique sensitive to the microstructures of biological tissue and assess water diffusion in the brain to provide insights into the characteristics of white matter.

White matter is a network of nerve fibers which are covered by myelin sheath that gives the white color. It connects neurons in different brain regions.

Grey matter contains the neuronal cell bodies, dendrites, and synapses. It has a role in information processing and human functioning.

Mindfulness-based stress reduction (MBSR) training is an eight-week program that contains meditation and body awareness to teach people how to cope with stress and anxiety.

Superior longitudinal fasciculus (SLF) is one of the major white matter tracts that connects the posterior part of the brain with the prefrontal cortex, and has an essential role in higher brain functions.

Corpus callosum is a thick nerve tract that connects the left and right side of the brain and enables communication.

Cortisol levels is a stress hormone which is produces and released by adrenal glands.

Fractional anisotropy (FA) represents the degree of directionality within a fiber track that is influenced by various aspects of the axonal microstructure, such as alterations in myelination and axon size.

METHODS

Mini-International Neuropsychiatric Interview (M.I.N.I) is a short clinical interview in order to diagnose psychiatric disorders.

Perceived Stress Scale (PSS) is validated, and well-established self-report scale for measuring the degree to which situations are perceived as stressful in the subject’s life.

Mindful Attention Awareness Scale (MAAS) is designed to measure an individual's focused awareness on what is occurring in the present moment.

State and Trait Anxiety Inventory (STAI) is a questionnaire to measure individuals' both stable (like a trait) and temporary (like feelings) aspects of anxiety.

Flow Short Scale (FSS) measures individuals` flow state like fluency of performance and absorption by activity.

RESULTS

Superior frontal gyrus (SFG) is the superolateral surface of the frontal lobe.

Brodmann area 31 (BA 31) is the dorsal posterior cingulate area.

Right uncinate fasciculus (rUNC) is a white matter tract that connects the prefrontal cortex with the right hippocampus.

DISCUSSION

Flanker Task is an attention task that measures the ability to suppress responses.

The Limbic system consists of several brain areas such as hippocampus, amygdala, hypothalamus, and cingulate cortex. It is involved in emotion, memory, and behavior.

Voxel-based morphometry is a MR-based computational technique that compares the brain regions by using voxel-wise estimations.
